# Reproducibility of radiomics quality score: an intra- and inter-rater reliability study

**DOI:** 10.1007/s00330-023-10217-x

**Published:** 2023-09-21

**Authors:** Tugba Akinci D’Antonoli, Armando Ugo Cavallo, Federica Vernuccio, Arnaldo Stanzione, Michail E. Klontzas, Roberto Cannella, Lorenzo Ugga, Agah Baran, Salvatore Claudio Fanni, Ekaterina Petrash, Ilaria Ambrosini, Luca Alessandro Cappellini, Peter van Ooijen, Elmar Kotter, Daniel Pinto dos Santos, Renato Cuocolo, for the EuSoMII Radiomics Auditing Group

**Affiliations:** 1 Institute of Radiology and Nuclear Medicine, Cantonal Hospital Baselland, Liestal, Switzerland; 2 Division of Radiology, Istituto Dermopatico dell’Immacolata (IDI) IRCCS, Rome, Italy; 3 Institute of Radiology, University Hospital of Padova, Padua, Italy; 4 Department of Advanced Biomedical Sciences, University of Naples “Federico II”, Naples, Italy; 5 Department of Medical Imaging, University Hospital of Heraklion, Crete, Greece; 6 Department of Radiology, School of Medicine, University of Crete, Heraklion, Crete Greece; 7 Section of Radiology, Department of Biomedicine, Neuroscience and Advanced Diagnostics (BiND), University of Palermo, Palermo, Italy; 8 MVZ Diagnostikum Berlin Gmbh, Diagnostisches Zentrum, Berlin, Germany; 9 Department of Translational Research, Academic Radiology, University of Pisa, Pisa, Italy; 10 Radiology Department, Research Institute of Children Oncology and Haematology of National Medical Research Center of Oncology n.a.N.N. Blokhin of Ministry of Health of RF, Moscow, Russia; 11 Department of Biomedical Sciences, Humanitas University, Pieve Emanuele, Milan, Italy; 12 Department of Radiation Oncology, University of Groningen, University Medical Center Groningen, Groningen, The Netherlands; 13 Department of Radiology, University Medical Center Freiburg, Freiburg, Germany; 14 Department of Radiology, University Hospital of Cologne, Cologne, Germany; 15 Department of Radiology, University Hospital of Frankfurt, Frankfurt, Germany; 16 Department of Medicine, Surgery and Dentistry, University of Salerno, Baronissi, Italy

**Keywords:** Reproducibility of results, Artificial intelligence, Radiomics, Inter-observer variability, Intra-observer variability

## Abstract

**Objectives:**

To investigate the intra- and inter-rater reliability of the total radiomics quality score (RQS) and the reproducibility of individual RQS items’ score in a large multireader study.

**Methods:**

Nine raters with different backgrounds were randomly assigned to three groups based on their proficiency with RQS utilization: Groups 1 and 2 represented the inter-rater reliability groups with or without prior training in RQS, respectively; group 3 represented the intra-rater reliability group. Thirty-three original research papers on radiomics were evaluated by raters of groups 1 and 2. Of the 33 papers, 17 were evaluated twice with an interval of 1 month by raters of group 3. Intraclass coefficient (ICC) for continuous variables, and Fleiss’ and Cohen’s kappa (*k*) statistics for categorical variables were used.

**Results:**

The inter-rater reliability was poor to moderate for total RQS (ICC 0.30–055, *p* < 0.001) and very low to good for item’s reproducibility (*k* − 0.12 to 0.75) within groups 1 and 2 for both inexperienced and experienced raters. The intra-rater reliability for total RQS was moderate for the less experienced rater (ICC 0.522, *p* = 0.009), whereas experienced raters showed excellent intra-rater reliability (ICC 0.91–0.99, *p* < 0.001) between the first and second read. Intra-rater reliability on RQS items’ score reproducibility was higher and most of the items had moderate to good intra-rater reliability (*k* − 0.40 to 1).

**Conclusions:**

Reproducibility of the total RQS and the score of individual RQS items is low. There is a need for a robust and reproducible assessment method to assess the quality of radiomics research.

**Clinical relevance statement:**

There is a need for reproducible scoring systems to improve quality of radiomics research and consecutively close the translational gap between research and clinical implementation.

**Key Points:**

*• Radiomics quality score has been widely used for the evaluation of radiomics studies.*

*• Although the intra-rater reliability was moderate to excellent, intra- and inter-rater reliability of total score and point-by-point scores were low with radiomics quality score.*

*• A robust, easy-to-use scoring system is needed for the evaluation of radiomics research.*

**Supplementary Information:**

The online version contains supplementary material available at 10.1007/s00330-023-10217-x.

## Introduction

Radiomics is an analysis tool to extract information from medical images that might not be perceived by the naked eye [1]. Over the course of a decade, several thousand studies have been published spanning diverse imaging disciplines in the field of radiomics research [2]. Nevertheless, the inherent complexity of these advanced methods that are employed to extract quantitative radiomics features may make it difficult to understand all facets of the analysis and evaluate the research quality, let alone to implement these published techniques in the clinical setting [3]. It is evident that easily applicable and robust tools for assessing the quality of radiomics research are needed to move the field forward.

With the aim of improving the quality of radiomics research methods, Lambin et al [4] proposed in 2017 an assessment tool, the radiomics quality score (RQS). Following the ideal workflow of conducting radiomics research, the RQS breaks it down into several steps and aims to standardize them. As a result, the RQS includes 16 items covering the entire lifecycle of radiomics research. Since its introduction in 2017, it has been widely adopted by the radiomics research community, and numerous systematic reviews using this assessment tool have been published [5–9]. However, it can still be inherently challenging for researchers or reviewers to correctly interpret and implement RQS and, therefore, assign scores, which are reproducible; as a result, most of the time the RQS scores are defined with a consensus decision and without a reproducibility analysis in these systematic reviews [5–7, 10–13]. Importantly, no intra- or inter-rater reproducibility analysis was presented in the original RQS publication [4].

According to a recent review article on systematic reviews using the RQS, in most cases the RQS is being used in a consensus approach: 27 out of 44 review articles chose to use consensus scoring, 10 did not even specify how the final scores were obtained, and only 7 of them used intraclass correlation coefficients (ICC) or kappa (*k*) statistics to assess inter-rater reliability [5]. Despite the positive connotation of a consensus decision, this does not necessarily mean that a score reached by consensus is reproducible. A consensus decision might solely reflect the most experienced rater, as novice voices could be suppressed, resulting in an underestimation of disagreement [14]. The decision to use consensus rather than inter-rater reliability could also presumably be due to challenges in applying the RQS and because ratings cannot be reliably reproduced across raters. Evidently, there is room for improvement in establishing an easily usable and reproducible tool for all researchers.

In this study, we aim to perform a large multireader study to investigate the intra- and inter-rater reliability of the total RQS score and individual RQS items. We believe that a robust method for assessing the quality of radiomics research is essential to carry the field into the future of radiology, rather than ushering in a reproducibility crisis.

## Material and methods

The study was conducted in adherence to the Guidelines for Reporting Reliability and Agreement Studies (GRRAS) reporting guidelines [15].

### Paper selection

**Fig. 1:**
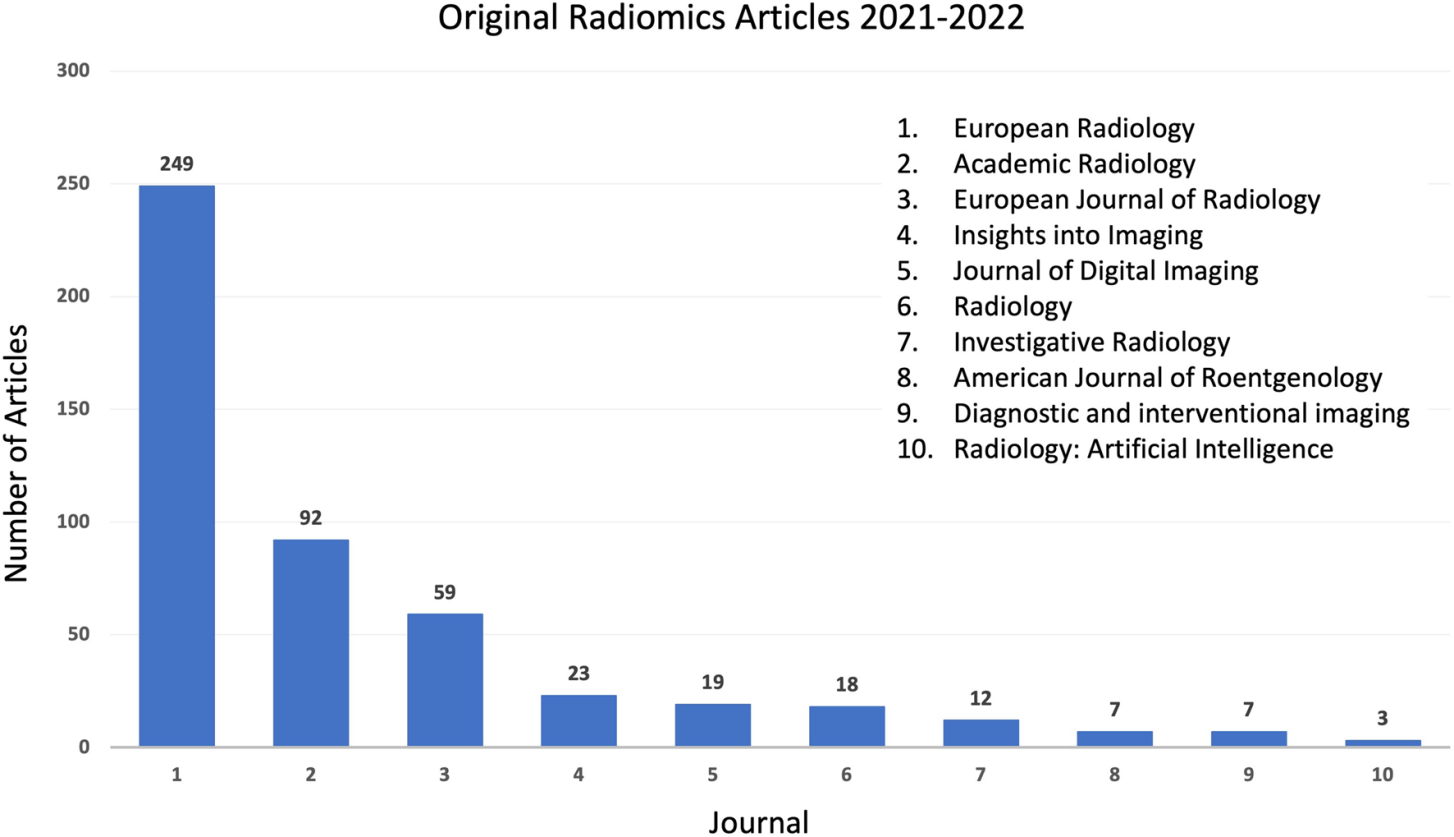
Bar graphs show the number of original radiomics articles published in first-quartal general radiology journals between 2021 and 2022

We included studies published recently in *European Radiology*, within an arbitrarily chosen period of 1 month until the start of our study. The following search query is used: (“European Radiology”[Journal]) AND (“radiomics”[Title/Abstract] OR “radiomic”[Title/Abstract]) AND (2022/09/01:2022/10/20[Date—Publication]). *European Radiology* was selected because it is a first-quartile (Q1—Scimago Journal Ranks) journal with the highest number of radiomics publications among all radiology journals; e.g., a PubMed search with keyword “radiomics” or “radiomic” in article title/abstract returns 249 original radiomics articles between January 1, 2021, and December 31, 2022 (Fig. 1).

**Fig. 2:**
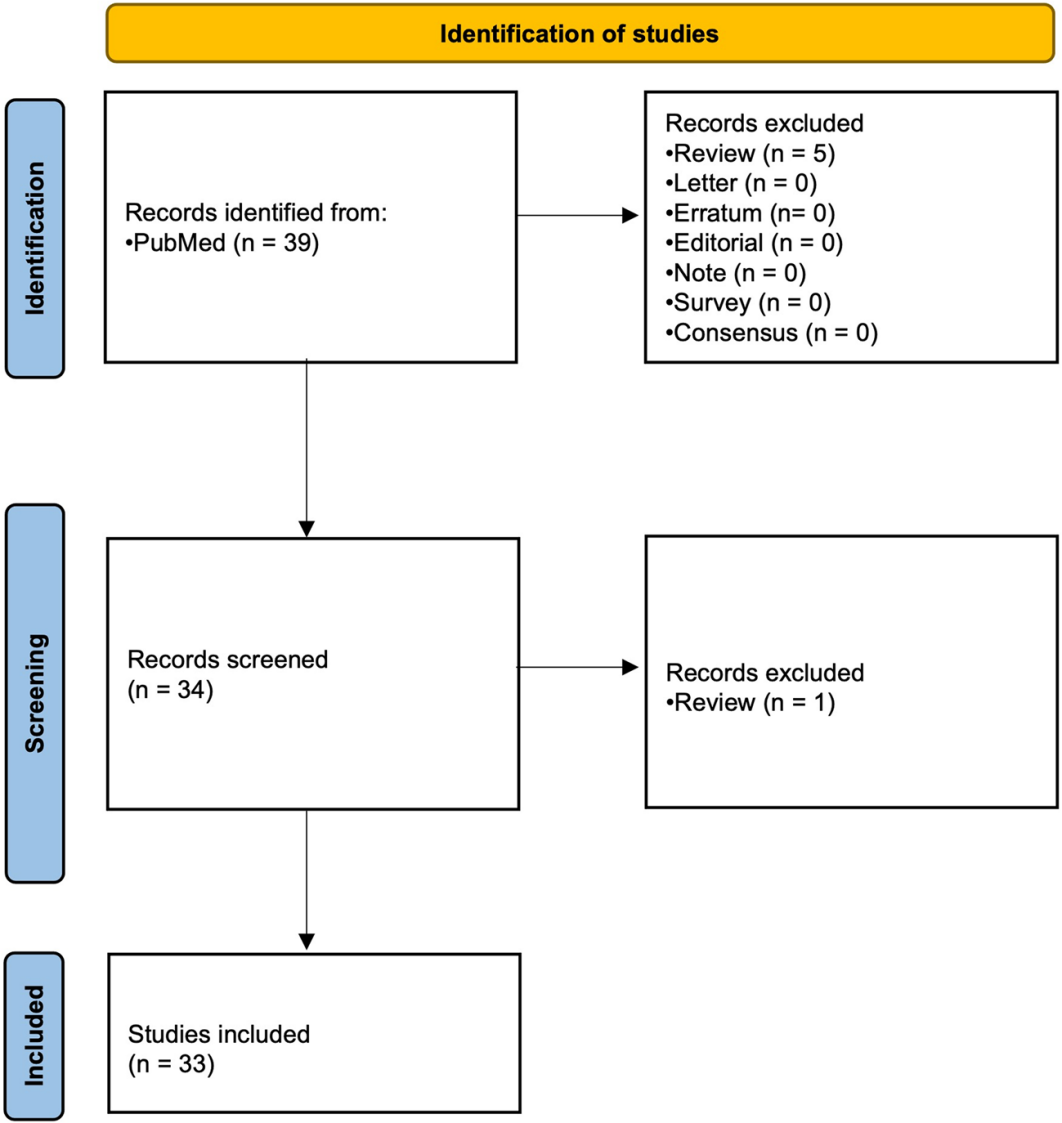
Study flow

We only included original research articles and excluded systematic reviews, literature reviews, editorials, letters, and corrections. After applying the inclusion and exclusion criteria, a total of 33 articles were selected for the study, which was above the minimum required sample size, i.e., 30, for the inter-rater reliability studies based on Guideline of Selecting and Reporting Intraclass Correlation Coefficients for Reliability Research (Fig. 2) [16].

### Rater selection and raters’ survey

A total of 9 raters with different backgrounds and experience levels were recruited for the study with an open call within the European Society of Medical Imaging Informatics (EuSoMII) Radiomics Auditing Group. They all completed a survey initially, which was sent to all raters by email to determine their level of expertise in the RQS application as well as the level of expertise in their occupation. Then, they were randomly assigned to the following groups according to their level of expertise: two inter-rater reliability groups, including one with and one without a training session on the use of RQS, and one intra-rater reliability group (Table 1).

**Table 1:** Rater characteristics according to the level of RQS rating experience

Rater	RQS rating experience^1^	Group^2^	Occupation	Years of experience^3^
1 (F.V.)	Novice	2	Radiologist	4
2 (I.A.)	Novice	1	Radiology resident	4
3 (E.A.P)	Intermediate	3	Radiologist	9
4 (S.C.F.)	Intermediate	2	Radiology resident	4
5 (A.B.)	Intermediate	1	Radiologist	8
6 (R.Ca.)	Intermediate	3	Radiologist	3
7 (L.U.)	Advanced	2	Radiologist	5
8 (M.K.)	Advanced	1	Radiology resident	3
9 (A.S.)	Advanced	3	Radiologist	4

^1^Novice: I have no previous experience, intermediate: I have some experience with RQS (e.g., 1–2 RQS evaluation), advanced: I have extensive experience with RQS (e.g., 3 or more RQS evaluation)

^2^Group 1: inter-rater reliability w/ training, group 2: inter-rater reliability w/o training, group 3: intra-rater reliability

^3^In occupation

**Fig. 3:**
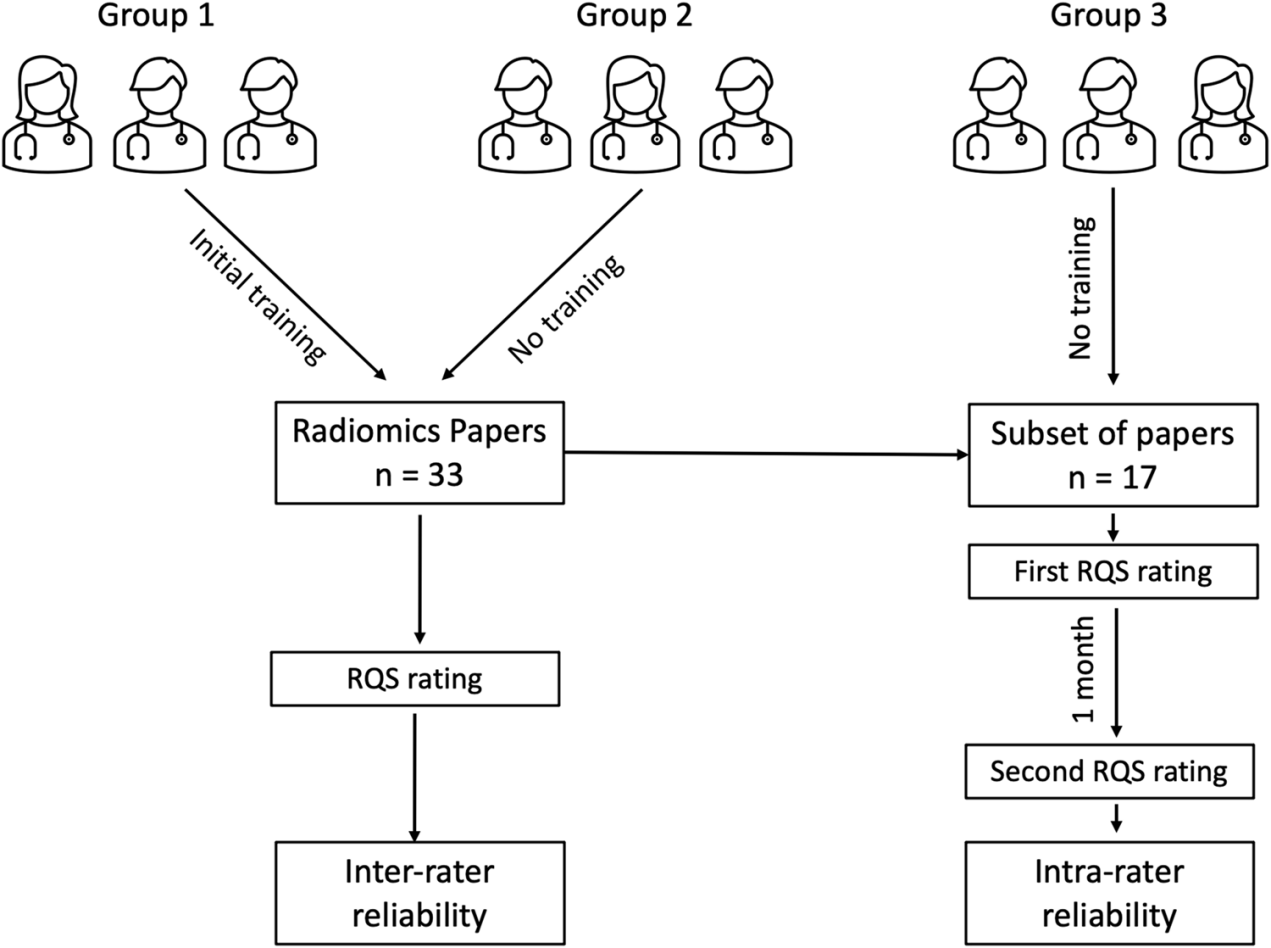
Study pipeline showing the different groups and their pathways in the study

The inter-rater reliability group with training (group 1) received a brief training session for the RQS assessment, during which they were instructed by an experienced rater (T.A.D.) about how to rate all items on a random article [17], and then, they separately completed the assessment of all 33 papers. The inter-rater reliability group without training (group 2) received no training at all on RQS and completed the ratings of all 33 papers. The intra-rater reliability group (group 3) received no training and was asked to score 17 out of 33 selected papers twice 1 month apart to minimize recall (Fig. 3). All raters provided their ratings as they read the article and their available supplementary material. A keyword search was also allowed if needed.

At the end of the study, raters received another survey to investigate the challenges they faced during the RQS assessment and their possible solutions.

### Statistical analysis

We used ICC (two-way, single rater, agreement, random effects model) for continuous variables, i.e., total RQS, and Fleiss’ and Cohen’s *k* statistics for categorical variables, i.e., item scores, as recommended [15, 16, 18]. Cohen’s *k* does not support to do comparisons of more than two raters/ratings, and Fleiss’ *k* should be used if there are more than two raters/ratings [19]. Therefore, Cohen’s kappa is used when there are two ratings/raters, i.e., group 3, and Fleiss’ kappa is used when there are more than two ratings/raters, i.e., groups 1 and 2, to compare [19]. We used two one-sided *t*-tests (TOST), a test of equivalence based on the classical *t*-test, to investigate group differences between mean RQS scores [20]. All statistical analysis was carried out with R software (version 4.1.1) and the “irr” and “TOSTER” packages were used [21].

## Results

### Paper selection

A total of 33 papers were included in this study. Two papers were technical papers, i.e., phantom studies, and all others were original research articles. The characteristics of included studies are shown in Table 2.

**Table 2:** Characteristics of included papers

Paper	First author	Journal	Publication year	Model utility	Body region	Sample size	Modality	Mean RQS^1^	Self-reported RQS
1	Noortman WA [22]	Eur Radiol	2022	Classification	Abdomen	38	PET-CT	10.3	N/A
2	Bao D [23]	Eur Radiol	2022	Prognostication	Head and neck	216	MRI	16	N/A
3	Chen Q [24]	Eur Radiol	2022	Detection and prognostication	Thorax	240	CT	15.2	N/A
4	von Schacky CE [25]	Eur Radiol	2022	Classification	Musculoskeletal	880	X-ray	13.2	N/A
5	Chu F [26]	Eur Radiol	2022	Prognostication	Abdomen	434	MRI	14.7	N/A
6	Xiang F [27]	Eur Radiol	2022	Prognostication	Abdomen	204	CT	14.8	N/A
7	Zhang H [28]	Eur Radiol	2022	Classification	Abdomen	138	CT	12.7	N/A
8	Zheng Y [29]	Eur Radiol	2022	Classification	Head and neck	388	CT	13	N/A
9	Lin M [30]	Eur Radiol	2022	Detection	Head and neck	489	US	12.5	N/A
10	Jiang J [31]	Eur Radiol	2022	Detection	Neurovascular	403	CT	14.3	N/A
11	Kang JJ [32]	Eur Radiol	2022	Detection	Neuro	149	MRI	8.7	N/A
12	Zhang D [33]	Eur Radiol	2022	Classification	Abdomen	209	MRI	13.7	N/A
13	Ma X [34]	Eur Radiol	2022	Classification	Thorax	612	CT	13.2	N/A
14	Li MD [54]	Eur Radiol	2022	Detection	Technical	108	US	2.5	N/A
15	Xie X [35]	Eur Radiol	2022	Classification	Neuro	89	MRI	8.5	N/A
16	Zhu C [36]	Eur Radiol	2022	Prognostication	Abdomen	106	CT	13.2	N/A
17	Fan Y [51]	Eur Radiol	2022	Detection	Thorax	192	MRI	14.8	27
18	Zhao M [37]	Eur Radiol	2022	Prognostication	Thorax	421	PET-CT	11.2	N/A
19	Frood R [38]	Eur Radiol	2022	Prognostication	Whole body	289	PET-CT	8.5	N/A
20	Zheng Q [39]	Eur Radiol	2022	Detection	Neuro	1650	MRI	8.8	N/A
21	Zhong J [40]	Eur Radiol	2022	Detection and prognostication	Musculoskeletal	144	MRI	14	N/A
22	Cheng B [41]	Eur Radiol	2022	Detection	Thorax	636	CT	14.8	N/A
23	Bi S [42]	Eur Radiol	2022	Prognostication/classification	Head and neck	128	MRI	13	N/A
24	Si N [43]	Eur Radiol	2022	Detection and classification	Cardiovascular-thorax	105	CT	12.3	N/A
25	Eifer M [44]	Eur Radiol	2022	Classification	Thorax	99	PET-CT	6	N/A
26	Chen H [45]	Eur Radiol	2022	Detection and classification	Neuro	609	MRI	11.3	N/A
27	Zhong J [55]	Eur Radiol	2022	Detection	Technical	N/A	CT	4.5	N/A
28	Zhang X [46]	Eur Radiol	2022	Prognostication	Thorax	172	CT	14.2	N/A
29	Zhang H [52]	Eur Radiol	2022	Detection	Neuro	355	CT	12.8	23
30	Zheng YM [47]	Eur Radiol	2022	Prognostication	Head and neck	217	CT	15.8	N/A
31	Salinas-Miranda E [48]	Eur Radiol	2022	Detection	Abdomen	122	CT	9.3	N/A
32	Nagaraj Y [49]	Eur Radiol	2022	Detection	Thorax	2720	CT	14.3	N/A
33	Bleker J [50]	Eur Radiol	2022	Detection	Abdomen	524	MRI	11.7	N/A

^1^According to the ratings of 6 raters from groups 1 and 2

### Rater selection and raters’ survey

Raters were randomly assigned to groups based on the initial survey results (Table 1). After completing the assessments, raters were given another survey to explore the challenges they faced during the RQS assessment and their possible solutions. All responses can be found in Table E1. One of the main problems they faced was the confusion caused by the lack of clear explanations of the RQS items in the main RQS paper and in the RQS checklist [4]. A list of the major issues with RQS along with our recommendations for a simpler approach is presented in Table 3.

**Table 3:** The potential reasons for challenges and proposed amendments for radiomics quality score break down by items

Item no	Topic	Score range	Challenges	Recommendation
1	Image protocol quality	+ 1 or + 2	Although this item looks straightforward, it is not exactly clear what authors meant by public protocol; is it an imaging protocol recommended by international guidelines or is it using previously validated protocol?	It is important to explicitly report image protocol to maintain feature reproducibility; however, it should also be defined how much detail will be necessary to ensure quality. We also encourage sharing image protocol explicitly, however, setting a bar for how many details should be presented so that the point could be assigned, and also it should be stated that the imaging protocol should ideally be agreed upon by the radiomics scientific community
2	Multiple segmentation	+ 1	Sole multiple segmentation does not ensure quality; multiple segmentations should always include reproducibility testing to be an added value It is difficult to justify this item since nowadays full automated segmentation algorithms, e.g., nnU-Net, are in use	Manual segmentation is rarely used but if there is multiple segmentation, we recommend assigning a point only if study includes a reproducibility testing We also recommend adapting this item to recent developments and including semiautomatic and automatic segmentation in the rating
3	Phantom study	+ 1	Most of the time, the studies are either phantom study dealing with feature reproducibility or clinical study which does not have any phantom. This item generates a clutter and is not suitable for neither study	We think that quality of phantom studies could be gauged neither by this item nor by total RQS, since most of the time phantom studies will get lower total score as we also observed in our study. Therefore, we recommend removing this item from the scoring system
4	Imaging at multiple time points	+ 1	Most of the times, this was not fulfilled, and even if it is, does not always ensure the quality of the study. Furthermore, this item could easily be misinterpreted and was therefore not reproducible as no clear definition was provided in the checklist as well as in the original RQS article	This item should be clearly explained
5	Feature reduction or adjustment for multiple testing	− 3 or + 3	Giving a minus score is confusing; moreover, it might cause problems while calculating the total score, people could forget using minus, or it could be mistakenly deleted during the analysis	We acknowledge that RQS works with reward and penalize system, but we recommend always assigning a positive score or giving no score at all
6	Multivariable analysis with non-radiomics features	+ 1	This item was confusing and it needs more clarification what non-radiomics features entail	We recommend adding clear explanation about non-radiomics features (i.e., standard of care clinical features or semantic imaging features). It should also be denoted if those non-radiomics features are included in the feature selection process or in the final model. Is the model holistic or if non-radiomics features are completely removed during feature selection?
7	Detect and discuss biological correlates	+ 1	This was the most confusing item according to all raters as most of the researchers superficially claim that their results are correlated with biologic features	We think this item needs clarification
8	Cut-off analyses	+ 1	Even when included in the analysis, the cut-off analysis does not necessarily ensure quality and it brings an arbitrary dichotomization to the table	It is not always necessary to conduct a cut-off analysis
9	Discrimination statistics	+ 1 or + 2	Some raters may be inexperienced	Giving some examples could be helpful
10	Calibration statistics	+ 1 or + 2	If models do not produce probabilistic outputs, then they are automatically penalized	Needs some adjustment for non-probabilistic outputs
11	Prospective study with registration to database	+ 7	Some studies, although retrospective, include prospectively collected test/validation set. This does not fulfill the requirements of being a prospective study	It should be specified that prospective data collection should be performed explicitly for radiomics clinical trials (i.e., not assigned for retrospective analyses of prospectively collected patients for other studies) and registered in the clinical trial database
12	Validation	− 5 to + 5	It is very confusing for the raters since there are several steps that should be defined	We think the most important component of a validation is internal and external validation. And internal validation step is sometimes fulfilled with a cross-validation. Therefore, we recommend scoring only these validation steps and keeping the range of the score + 1 to + 2, one point for each step. Moreover, an independent validation study should be rated the same as external validation
13	Comparison to reference standard	+ 2	This was one of the most confusing items since most of the studies either does not fulfill this step or there is a lack of clearly defined reference standard	We recommend providing a clear definition of reference standard and outcomes
14	Potential clinical utility	+ 2	This item is very open ended and most of the time authors tend to mention great potential of clinical utility even though their decision curve analysis shows opposite	We recommend abolishing this item and instead accepting external validation as a sign of potential clinical utility
15	Cost-effectiveness analysis	+ 1	Almost none of the studies provides this since majority studies are exploratory and there is still lack of prospective studies and RCTs	This item would be unnecessary for usual radiomics studies and might be more suitable for RCTs or radiation oncology studies. We recommend omitting this item
16	Open science and data	+ 1 to + 4	Different incremental steps create a confusion	We think this is very important, but we think scoring system must be simplified. Rating the open data, open code, open model would have been enough
Total points	The total score is often converted into percentage values	− 8 to + 36	When there are negative results, it is difficult to convert them to percentages. A paper with a score of − 8 will score the same in percentage as a paper with a score of 0. So, we can assume that both papers are of low quality, but with a negative score, the magnitude of this “lowness” is difficult to understand Moreover, it is hard to understand when there is no reference point proposed regarding quality	We recommend using only 0 or positive values for scoring. Also, along with the total score we propose implementation of thresholds: 0–25% low, 25–50% average, 50–75% moderate, 75–100% excellent

### Statistical analysis

#### Inter-rater reliability

The inter-rater reliability was poor between raters of group 1 (ICC 0.30; 95% CI [0.09–0.52]; *p* = 0.0015), and moderate between raters of group 2 (ICC 0.55; 95% CI [0.29–0.74]; *p* < 0.001), and remained low-to-moderate when comparing raters of groups 1 and 2 with the same level of experience (ICC 0.26–0.61). This trend was observed also for intra-group reliability analysis: Raters of group 1 showed poor inter-rater reliability and raters of group 2 moderate inter-rater reliability (Table 4).

**Table 4:** Results of the intra- and inter-rater reliability analysis for overall RQS

	ICC	95% CI	*p*
Inter-rater analysis			
Overall ICC			
Group 1	0.301	0.09–0.52	0.0015
Group 2	0.549	0.29–0.74	< 0.001
Intra-group ICC			
Group 1			
Raters 2 vs 5	0.304	− 0.04 to 0.58	0.0418
Raters 2 vs 8	0.232	− 0.07 to 0.51	0.0685
Raters 5 vs 8	0.358	0.04–0.61	0.0137
Group 2			
Raters 1 vs 4	0.603	− 0.05 to 0.85	0.0398
Raters 1 vs 7	0.510	0.21–0.72	0.001
Raters 4 vs 7	0.529	0.17–0.75	< 0.001
Inter-group ICC (matched level of experience)			
Group 1 novice vs group 2 novice	0.255	− 0.06 to 0.54	0.0612
Group 1 intermediate vs group 2 intermediate	0.609	0.34–0.79	< 0.001
Group 1 advanced vs group 2 advanced	0.349	− 0.08 to 0.66	0.0649
Intra-rater analysis			
Group 3			
Rater 3	0.522	0.09–0.79	0.009
Rater 6	0.910	0.77–0.96	< 0.001
Rater 9	0.989	0.96–0.99	< 0.001

#### Intra-rater reliability

In the intra-rater reliability analysis, only rater 3, with intermediate experience level, showed moderate reliability between the first and second read (ICC 0.522; 95% CI [0.09–0.79]; *p* = 0.009), whereas rater 6 and rater 9, with advanced experience level, showed excellent intra-rater reliability (ICC 0.91; 95% CI [0.77–0.96]; *p* < 0.001 and 0.99; 95% CI [0.96–0.99]; *p* < 0.001, respectively).

#### Reliability of RQS items’ score

The inter-rater reliability for RQS items’ score reproducibility within groups 1 and 2 was very low. The only items that had high inter-rater reliability were items 3 (phantom study) and item 15 (cost-effectiveness analysis). All other items had poor to moderate inter-rater reliability. The intra-rater reliability of RQS items’ score was higher and most of the items had moderate to good intra-rater reliability, if not perfect. The mean value and standard deviation of *k* values for group 1 was 0.18 ± 0.33, for group 2 was 0.43 ± 0.3, and within group 3 for rater 3 was 0.7 ± 0.3, rater 6 was 0.75 ± 0.22, and rater 9 was 0.88 ± 0.27. Fleiss’ *k* for each RQS item of groups 1 and 2 and Cohen’s *k* for each RQS item of group 3 are summarized in Table 5.

**Table 5:** Results of the intra- and inter-rater reliability analysis for RQS item reproducibility

	Group 1		Group 2		Group 3					
					Rater 3		Rater 6		Rater 9	
	k*	*p*	k*	*p*	*k*^+^	*p*	*k*^+^	*p*	*k*^+^	*p*
Item 1	0.03	0.79	0.32	< 0.001	0.46	0.008	0.83	< 0.001	− 0.03	0.79
Item 2	0.26	0.01	0.51	< 0.001	0.57	0.006	0.72	0.002	0.82	< 0.001
Item 3	1	0	1	< 0.001	1	< 0.001	1	< 0.001	1	< 0.001
Item 4	− 0.1	0.24	0.54	< 0.001	1	< 0.001	1	< 0.001	1	< 0.001
Item 5	0.0006	0.99	0.46	< 0.001	1	< 0.001	0.64	0.004	1	< 0.001
Item 6	0.35	< 0.001	0.55	< 0.001	0.41	0.09	1	< 0.001	1	< 0.001
Item 7	0.38	< 0.001	0.19	0.05	0.82	< 0.001	0.77	0.001	1	< 0.001
Item 8	− 0.16	0.11	0.52	< 0.001	0.01	0.94	0.36	0.09	1	< 0.001
Item 9	0.15	0.06	0.06	0.44	1	< 0.001	0.76	< 0.001	0.58	0.001
Item 10	0.75	< 0.001	0.56	< 0.001	0.37	0.04	0.79	< 0.001	1	< 0.001
Item 11	− 0.02	0.83	− 0.02	0.83	1	< 0.001	1	< 0.001	1	< 0.001
Item 12	0.22	< 0.001	0.43	< 0.001	0.59	< 0.001	0.80	< 0.001	0.89	< 0.001
Item 13	− 0.04	0.68	0.50	< 0.001	0.45	0.02	0.54	0.01	1	< 0.001
Item 14	0.23	0.01	0.22	0.02	0.76	< 0.001	0.59	0.01	0.85	< 0.001
Item 15	− 0.01	0.91	1	< 0.001	1	< 0.001	1	< 0.001	1	< 0.001
Item 16	− 0.21	< 0.001	0.03	0.72	1	< 0.001	0.30	0.20	1	< 0.001

^*^Fleiss’ *k*

^+^Cohen’s

Moreover, we found that two of the 33 manuscripts included a self-reported RQS which was higher than the scores assigned by the raters in our study as reported in Table 3 [51, 52].

**Fig. 4:**
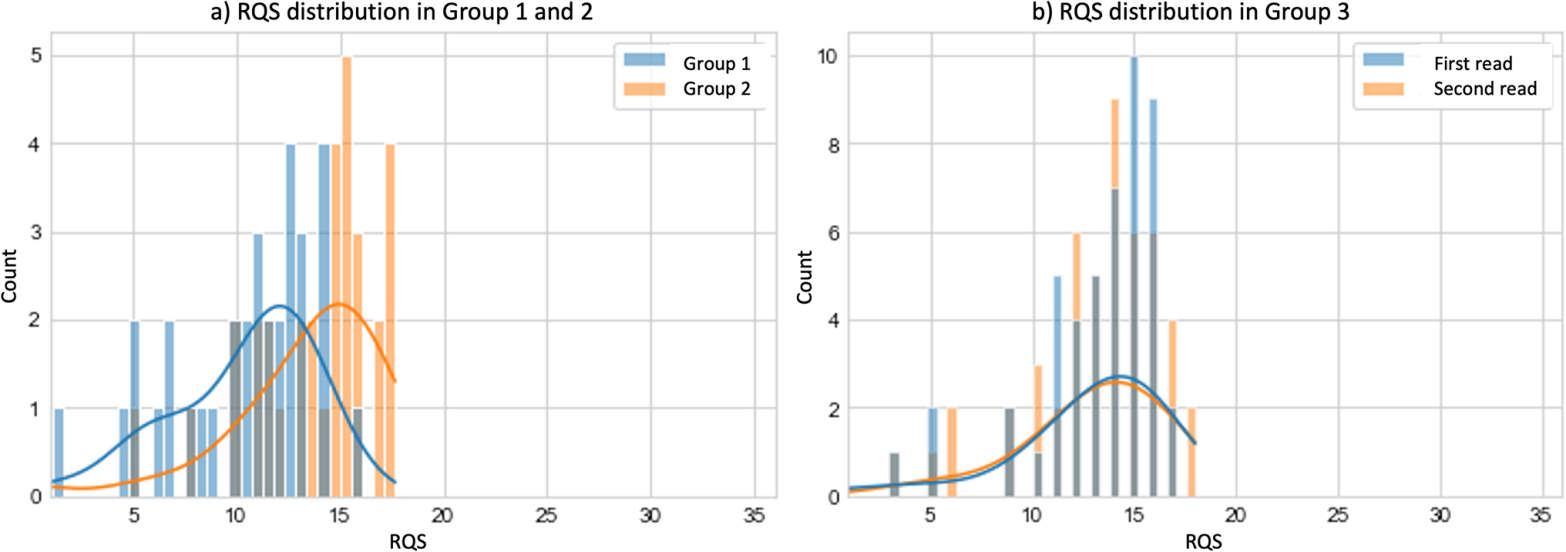
Histograms and kernel density estimation plots showing the overall distribution of mean RQS separately (**a**) in group 1 (depicted in blue) and group 2 (depicted in orange) and (**b**) in group 3 first read (depicted in blue) and second read (depicted in orange)

**Fig. 5:**
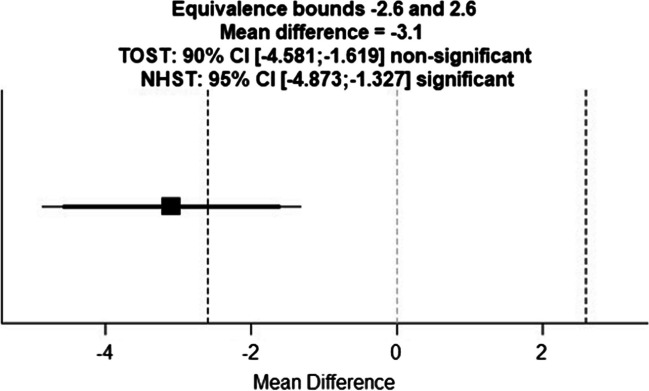
Two one-sided *t*-test graph

The mean RQS for group 1 was 10.2 ± 3.5 and for group 2 13.2 ± 4 and the mean RQS for group 3 first read was 12.23 ± 5 and second read was 12.4 ± 4.9 (Fig. 4). Two one-sided *t*-tests were applied between the mean RQS value obtained by readers of groups 1 and 2. The lower and upper bounds were calculated to have a statistical power of 0.8 with an alpha of 0.05. Thus, with a lower and upper equivalence bound of  ± 2.6 and a mean difference of  − 3.1, the *p* value for the lower bound was 0.7 and for the upper bound was  < 0.001 (Fig. 5).

## Discussion

In this study, we conducted a multireader study and investigated the intra- and inter-rater reliability of total RQS as well as individual RQS item scores, involving readers with different experience levels regarding RQS rating. We found that despite being widely adopted, the RQS tool is not straightforward to comprehend and adopt, and its results may not be reproducible in many cases (inter-rater reliability ICC 0.30–055, *p* < 0.001 and intra-rater reliability ICC 0.522, *p* = 0.009 for total RQS; inter-rater group *k* − 0.12 to 0.75 and intra-rater group *k* − 0.40 to 1 for item’s reproducibility). Our results suggest that there is room for improvement to establish an easy-to-use scoring framework for authors, reviewers, and editors to assess the quality of radiomics studies.

To date, RQS has served as a valuable tool to fill the gap for guidance on the quality assessment of radiomics research. Similarly to Lambin et al [4], we believe that the quality of radiomics research should not be compromised, and researchers should transparently report their methods to ensure quality and reproducibility. In addition, to further advance the field, researchers should be incentivized to adopt open science practices. Nonetheless, any questionnaire or score intended for the evaluation of research or clinical practices should be rigorously evaluated for its reliability and reproducibility. To date, this has not happened for RQS even though it is widely used as a tool to assess the quality of radiomics research. Therefore, we believe that 5 years after its introduction, the RQS system should be updated to be more easily used by researchers, reviewers, and editors. Recently, a new reporting guideline has been published that covers all requirements, which are necessary to improve radiomics research quality and reliability [53]. We think our recommendations are also in line with this new guideline.

Interestingly, we found slight negativity of the training session that took place prior to the RQS application (according to the two one-sided *t*-test, groups 1 and 2 were not equivalent and statistically different with a lower and upper equivalence bound of  ± 2.6 and a mean difference of  − 3.1, lower bound *p* value = 0.7, upper bound *p* < 0.001). The raters of group 1 showed poor inter-rater reliability despite the training and group 2 showed moderate inter-rater reliability even though they have not received any instructions beforehand. Moreover, we observed the positive effect of more experience only in the intra-rater reliability analysis. The advanced raters showed perfect intra-rater reliability results, whereas the less experienced rater had moderate reliability. We have not observed an effect of experience in the inter-rater reliability analysis.

The raters indicated that the RQS instructions were not self-explanatory in most cases; therefore, they needed more time to interpret the RQS items and consecutively to assign a score. For example, item 4, i.e., “imaging at multiple time points,” was one such item that had low inter-rater reproducibility (*k* =  − 0.1 in group 1; *k* = 0.54 in group 2) due to unclear item definition in the checklist as well as in the article [4]. It could be argued that this refers to imaging at different time points within the same examination, i.e., imaging in the arterial/portal venous phase; inspiration/expiration; and test-retest. On the other hand, it could also be argued that this is a hint to longitudinal studies where imaging is performed at different time points, i.e., within 3 months, to perform a delta radiomics analysis. Also, the non-standard range of values, i.e., the sudden change from  + 1 to  + 2 to  − 7 to  + 7, caused confusion for the authors when assigning a score, without a proper justification of such non-standard range (e.g., for items 5, 12, and 16). A non-standard range would have been acceptable in the case of weighting the item scores according to their importance (Table 3).

One of the problems was that some of the items that may be unusual for the radiology workflow led to confusion instead of clarity. For example, some of the radiomics studies deal only with phantoms with an intention to cover technical aspects or to test the stability of radiomics features [54, 55]. In this case, an item dealing with phantom studies (item 3) might be a good idea, but in practice, clinical radiomics studies do not necessarily use this phantom step to stabilize their features and do not fulfill this item. Although the transferability of feature robustness from a phantom to a specific biological tissue in the setting of radiomics should still be demonstrated, technically focused phantom studies typically lack clinical validation and therefore tend to achieve lower scores in the RQS system. Similar issues were identified with item 15, which addresses cost-effectiveness analysis. This is very unusual for current radiomics studies, i.e., mostly retrospective, and rarely prospective let alone being included in a randomized controlled study. Also, the definition of cost for radiomics still represents a challenge and, to the best of our knowledge, no published cost-effectiveness analysis for radiomics exists in the literature [56]. Its value in terms of methodological quality could benefit from more research on the topic. Although items 3 and 15 were the most reproducible (Table 5), we argue that they create unnecessary clutter and had a limited impact on overall study quality, as they tended to be always absent (i.e., item 3 and item 15) based exclusively on the study aim or design.

Nowadays, more and more studies utilize deep learning for radiomics analysis; however, the current RQS tool mainly focuses on hand-crafted radiomics, and items specifically addressing the methodological challenges typical to deep learning approaches on radiomics are lacking. Consequently, robust and properly designed deep learning studies might be penalized with a low RQS total score merely because they fail to address questions that are relevant to deep learning methodology. Moreover, in the current RQS tool, sample size analysis or properly selecting the subjects is not rated. We think that sample size analysis and defining the study subjects could be included since study design is one of the most critical steps of a study [57].

We noted that some of the studies included self-reported scores in their publications, but, unfortunately, we found these to be an overly enthusiastic assessment, and observed a large discrepancy when compared with mean RQS results from our multireader analysis [51, 52]. It is not a new phenomenon that researchers tend to overestimate their results and report them within a rose-tinted frame of enthusiasm. This is just a cautionary note for reviewers, editors, and readers to aid correct evaluation of self-reported RQS scores based on our evidence.

Our study had some limitations. We only included a limited amount of papers, but according to the guidelines, it is still more than the minimum required sample size for the inter-rater reliability studies [16]. Moreover, we included articles only from *European Radiology*. However, in the field of medical imaging, *European Radiology* is the Q1 journal with the highest number of radiomics publications over the past 2 years, ensuring the quality of the studies from a selection of diverse radiomics research areas. In addition, although we intended to explore the effects of training in our study, we did not find any positive effects of training on the reproducibility of RQS. On the one hand, using only one paper as a teaching example might not be sufficient to capture a significant difference. On the other hand, a tool that requires extensive training, even among researchers in the field, to reach adequate reproducibility reveals the limitations of the RQS. Moreover, we have not investigated the effect of training for inter-rater reliability analysis; however, we think the effect of training might be too small to detect as we already found that the intra-rater reliability was moderate to excellent.

In conclusion, we have come a long way in the field of radiomics research, but on the long road to clinical implementation, we need reproducible scoring systems as much as we need reproducible radiomics research. We hope that our recommendations for a more straightforward radiomics quality assessment tool will help researchers, reviewers, and editors to achieve this goal.

### Supplementary Information

Below is the link to the electronic supplementary material.

Supplementary file1 (PDF 179 KB)

## Abbreviations

EuSoMII: European Society of Medical Imaging Informatics
GRRAS: Guidelines for Reporting Reliability and Agreement Studies
ICC: Intraclass coefficient
k: Kappa statistics
Q1: First quartal
RQS: Radiomics quality score
TOST: Two one-sided *t*-tests

## Declarations

### Guarantor

The scientific guarantor of this publication is Renato Cuocolo.

### Statistics and biometry

Armando Ugo Cavallo (co-author) and Renato Cuocolo (co-author) have significant statistical expertise.

### Methodology

• Retrospective

• Observational

• Multicenter study
