# Supplementary material for: Reproducibility of radiomics quality score: an intra- and inter-rater reliability study
Source: Eur Radiol. 2023 Sep 21;34(4):2791–804. doi: 10.1007/s00330-023-10217-x (PMC10957586; doi:10.1007/s00330-023-10217-x)
Supplement: Supplementary file 1 — Supplementary file1 (PDF 179 KB) [file 330_2023_10217_MOESM1_ESM.pdf]

Reproducibility of radiomics quality score: an intra- and inter-rater reliability study

Electronic Supplementary Material

Table E1. Table shows the final survey responses of all raters.

Rater	Item #1	Item #2	Item #3	Item #4	Item #5	Item #6	Item #7	Item #8	Item #9	Item #10	Item #11	Item #12	Item #13	Item #14	Item #15	Item #16
1 (F.V.)	1	1	1	1	1	1	1	1	2	3	1	1	2	1	2	2
2 (I.A.)	1	1	1	1	1	1	1	1	2	2	1	2	1	1	2	2
3 (E.A.P)	1	1	1	1	1	3	2	1	1	1	1	1	2	1	1	1
4 (S.C.F.)	1	1	1	1	1	1	1	3	2	2	1	2	1	2	2	1
5 (A.B.)	1	1	1	2	1	2	2	1	1	1	1	1	2	1	1	1
6 (R.Ca.)	1	1	1	2	2	2	2	2	2	3	1	1	3	2	2	3
7 (L.U.)	1	2	1	1	1	1	2	2	2	2	1	2	3	2	1	1
8 (M.K.)	2	1	1	1	1	1	2	1	1	1	1	2	1	1	1	1
9 (A.S.)	0	2	1	1	1	1	1	2	1	1	1	1	2	1	1	1

Item score key: 1. very clear and easily understandable. 1. a little challenging to understand. 3. very difficult to understand.

Eur Radiol (2023) Akinci D'Antonoli T, Cavallo AU, Vernuccio F et al.
